# Incidence of thyroid diseases: Results from the Brazilian Longitudinal Study of Adult Health (ELSA-Brasil)

**DOI:** 10.20945/2359-3997000000348

**Published:** 2021-04-12

**Authors:** Isabela M. Benseñor, José Augusto Sgarbi, Carolina Castro Porto Silva Janovsky, Bianca Almeida Pittito, Maria de Fátima Haueisen Sander Diniz, Maria da Conceição Chagas de Almeida, Sheila Maria Alvim, Sandhi M. Barreto, Luana Giatti, Bruce B. Duncan, Maria Inês Schmidt, Maria de Jesus M. Fonseca, Rosane H. Griep, Maria del Carmen B. Molina, José Geraldo Mill, Itamar de Souza Santos, Alessandra C. Goulart, Paulo A. Lotufo

**Affiliations:** 1 Universidade de São Paulo Centro de Pesquisa Clínica e Epidemiológica São Paulo SP Brasil Centro de Pesquisa Clínica e Epidemiológica, Universidade de São Paulo, São Paulo, SP, Brasil; 2 Faculdade de Medicina de Marília Divisão de Endocrinologia Marília SP Brasil Divisão de Endocrinologia, Faculdade de Medicina de Marília, Marília, SP, Brasil; 3 Universidade Federal de São Paulo Departamento de Medicina Preventiva São Paulo SP Brasil Departamento de Medicina Preventiva, Universidade Federal de São Paulo, São Paulo, SP, Brasil; 4 Universidade Federal de Minas Gerais Faculdade de Medicina Belo Horizonte MG Brasil Faculdade de Medicina, Universidade Federal de Minas Gerais (UFMG), Belo Horizonte, MG, Brasil; 5 Fundação Oswaldo Cruz – Fiocruz Instituto Gonçalo Moniz Salvador BA Brasil Instituto Gonçalo Moniz, Fundação Oswaldo Cruz – Fiocruz, Salvador, BA, Brasil; 6 Universidade Federal da Bahia Instituto de Saúde Coletiva Salvador BA Brasil Instituto de Saúde Coletiva, Universidade Federal da Bahia, Salvador, BA, Brasil; 7 Universidade Federal de Minas Gerais Medicina Preventiva Belo Horizonte MG Brasil Medicina Preventiva, Universidade Federal de Minas Gerais, Belo Horizonte, MG, Brasil; 8 Universidade Federal do Rio Grande do Sul Programa de Pós-Graduação em Epidemiologia Porto Alegre RS Brasil Programa de Pós-Graduação em Epidemiologia, Universidade Federal do Rio Grande do Sul, Porto Alegre, RS, Brasil; 9 Fundação Oswaldo Cruz Escola Nacional de Saúde Pública Departamento de Epidemiologia e Métodos Quantitativos Rio de Janeiro RJ Brasil Departamento de Epidemiologia e Métodos Quantitativos, Escola Nacional de Saúde Pública, Fundação Oswaldo Cruz, Rio de Janeiro, RJ, Brasil; 10 Fundação Oswaldo Cruz Instituto Oswaldo Cruz Laboratório de Educação em Saúde e Meio Ambiente Rio de Janeiro RJ Brasil Laboratório de Educação em Saúde e Meio Ambiente, Instituto Oswaldo Cruz, Fundação Oswaldo Cruz, Rio de Janeiro, RJ, Brasil; 11 Universidade Federal do Espírito Santo Departamento de Nutrição Vitória ES Brasil Departamento de Nutrição, Universidade Federal do Espírito Santo, Vitória, ES, Brasil; 12 Universidade Federal do Espírito Santo Departamento de Ciências Fisiológicas Vitória ES Brasil Departamento de Ciências Fisiológicas, Universidade Federal do Espírito Santo, Vitória, ES, Brasil

**Keywords:** Overt thyroid diseases, subclinical thyroid diseases, hyperthyroidism, hypothyroidism, incidence

## Abstract

**Objective:**

To evaluate incidence of subclinical and overt hyperthyroidism and hypothyroidism.

**Subjects and methods:**

The Brazilian Longitudinal Study of Adult Health (ELSA-Brasil) is a prospective cohort study of 15,105 civil servants, examined at baseline and over a 4-year follow-up. This analysis included 9,705 participants with normal thyroid function at baseline, follow-up information about thyroid function and with no report of using drugs that may interfere in the thyroid function. Thyroid function was defined by TSH/FT4 levels or routine use of thyroid hormones/anti-thyroid medications. Annual and cumulative (over 4-year) incidence rates were presented as percentages (95% Confidence Intervals).

**Results:**

The incidence of all overt and subclinical thyroid disease was 6.7% (1.73%/year): 0.19% for overt hyperthyroidism (0.048%/year), 0.54% for subclinical hyperthyroidism (0.14%/year), 1.98% for overt hypothyroidism (0.51%/year), and 3.99% for subclinical hypothyroidism (1.03%/year). The incidence of all thyroid diseases was higher in women, when compared to men, with a low women:men ratio (1.36). For Blacks the highest incidence was for overt hyperthyroidism, while for Whites, the highest incidence was for overt hypothyroidism. However, the highest incidence of overt hyperthyroidism was detected in Asian descendants. The presence of antithyroperoxidase antibodies at baseline was associated with higher incidence of overt thyroid diseases.

**Conclusion:**

These results showed a high incidence of hypothyroidism, which is compatible with a country with a more-than-adequate iodine intake. The low women:men ratio of the incidence of thyroid dysfunction highlights the importance of the diagnosis of thyroid diseases among men in Brazil.

## INTRODUCTION

Thyroid dysfunction is a very common disease in the general population worldwide ( 1 , 2 ). Although there are a considerable number of prevalence studies, data on the incidence of thyroid diseases is still scarce. Few studies have evaluated the incidence of thyroid diseases worldwide, such as subclinical and overt hyperthyroidism and hypothyroidism ( 3 , 4 ). In Europe, a recent meta-analysis of seven studies reported an incidence of thyroid diseases of 259.12 (254.39-263.9) events per 100,000 per year: 226.2 (222.26-230.17) and 51 (49.23-52.88) events per 100,000 per year for hypothyroidism and hyperthyroidism, respectively ( 3 ). One study from Iran, one of the few low- and middle-income countries with information about incidence of thyroid diseases and known to be iodine-sufficient, reported an incidence of hypothyroidism of 3.3 in women and 2.1 in men per 1,000 persons/year, while the incidence of hyperthyroidism was found to be 3.8 in women and no cases in men per 1,000 persons/year after a 6-year follow-up ( 4 ).

Epidemiological studies on the incidence of thyroid diseases are very important, because many patients are asymptomatic or have reported unspecific symptoms, which can lead to a high rate of underdiagnosis ( 1 , 2 , 4 ). Additionally, the incidence of thyroid disorders is related to the availability of iodine ( 5 , 6 ), selenium and other trace elements ( 7 ), chemical contaminants ( 8 ), frequency of autoimmune thyroid diseases ( 9 ), and genetic risk factors. ( 10 ) Incidence also varies widely in populations according to the area of residence. ( 5 - 7 ) Finally, as recently proposed, subclinical thyroid diseases can be considered a non-classical risk factor for coronary heart disease ( 11 ), being associated with all-cause mortality ( 12 - 14 ) and cardiovascular mortality ( 13 , 15 ).

In Brazil, some information can be found regarding the prevalence of thyroid diseases in population-based ( 16 , 17 ) and large epidemiological studies ( 18 ). The available data indicate rates within the highest prevalence of hypo- and hyperthyroidism in the world ( 19 ). However, to the best of our knowledge, no large study on the incidence of thyroid diseases in Brazil has been conducted to date. Therefore, the objective of this analysis is to evaluate the incidence of all overt and subclinical hypothyroidism and hyperthyroidism using data from the baseline and over a 4-year follow-up of the Brazilian Longitudinal studies of Adult Health (ELSA-Brasil).

## SUBJECTS AND METHODS

The Brazilian Longitudinal Study of Adult Health (ELSA-Brasil) is a prospective cohort study of civil servants, 35 to 74 years of age, in six cities in Brazil, examined at baseline (2008-2010) and after a 4-year follow-up (2012-2014). Although the study is focused on cardiovascular diseases and diabetes, together with associated factors, it also includes information about non-classical risk factors for cardiovascular disease, such as the subclinical thyroid function ( 20 , 21 ).

Briefly, all 15,105 participants are civil servants of six teaching and research institutions located in six different cities in Brazil: Salvador ( *Universidade Federal da Bahia* – UFBA, 2029 participants), Vitória ( *Universidade Federal do Espírito Santo* , 1055 participants), Belo Horizonte ( *Universidade Federal de Minas Gerais* , 3115 participants), Rio de Janeiro ( *Fundação Oswaldo Cruz* , 1784 participants), São Paulo ( *Universidade de São Paulo* – USP, 5061 participants), and Porto Alegre ( *Universidade Federal do Rio Grande do Sul* , 2061 participants). There are no important differences among sites. All participants are civil servants with higher education attainment and average monthly family net income compared to general population in Brazil. ELSA-Brasil sample is similar to people living in metropolitan areas in Brazil. Although the higher education attainment and average income, in all 6 centers, sample was selected according to 3 categories: non-skilled, technical and faculties with a clear socioeconomic gradient among them.

The sample size estimation was calculated to allow sex-specific analyses. The ELSA-Brasil protocol was approved at each of the six study centers by the local Institutional Review Board, which addresses research in human participants. All participants provided a signed informed consent (CAAE Number at Plataforma Brasil: 08109612.7.1001.0076). The second evaluation of the participants was performed between 2012 and 2014. In both examinations at baseline and follow-up each participant was interviewed and examined in the study research centers, following standard protocols developed for the study. Trained study staff conducted the interviews and examinations, following strict quality control procedures as previously described ( 22 , 23 ).

### Participants

**Figure 1:**
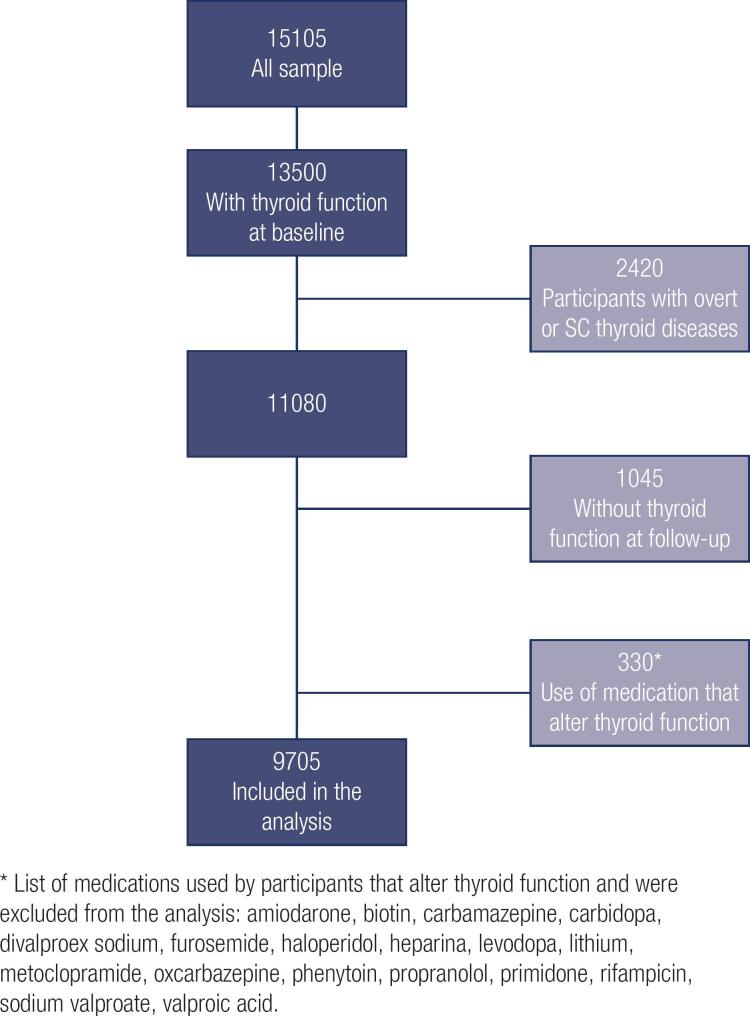
Participants included in the analysis.

This analysis included all participants with information about thyroid function at baseline and over the 4-year follow-up ( Figure 1 ). The main reasons for not having data on thyroid function in follow-up analyses were death or retirement with change of place of residence outside the metropolitan area of the Research Center and not being available to come to the clinical center for examination.

### Definition of thyroid function

All tests were done in the Central Laboratory localized at *Hospital Universitário* , USP, São Paulo, Brazil. Blood samples were drawn in each center, centrifuged to obtain serum for biochemistry and to determine hormone levels and aliquoted in cryotubes at -80°C. Each month, the samples were transported to São Paulo and the tests were done in the Central Laboratory. The Research Center of São Paulo was responsible for a centralized training of all local laboratory teams in São Paulo as well as under strict quality control of all tasks ( 24 ).

Thyrothropin (TSH) and free-thyroxine (FT4) levels were determined by a third-generation immunoenzymatic assay (Roche Diagnostics, Manheim, Germany). Thyroid dysfunction was defined by TSH and FT4 levels or by the routine use of thyroid hormones or anti-thyroid medications, such as propylthiouracil or thiamazole. Cut-off for normal TSH was 0.4-4.0 mIU/L and for normal FT4 was 0.93–1.7 ng/dL. Levels of anti-thyroperoxidase antibodies (TPOAb) were measured by electrochemiluminescence (Roche Diagnostics, Mannheim, Germany) and were considered positive when ≥34 IU/mL and negative as <34 IU/mL.

Based on TSH and FT4 levels and the use of medications to treat thyroid disorders, participants were categorized into the five following groups: ( 1 ) overt hyperthyroidism (low TSH, high FT4, or use of medications to treat hyperthyroidism), ( 2 ) subclinical hyperthyroidism (low serum TSH, normal FT4, and no use of thyroid drugs), ( 3 ) euthyroidism (normal TSH without use of thyroid medication), ( 4 ) subclinical hypothyroidism (high TSH, normal FT4 levels, and no use of thyroid drugs), and ( 5 ) overt hypothyroidism (high TSH, low FT4, or use of levothyroxine).

### Other baseline variables

Fasting plasma glucose was measured using a hexokinase method. Total cholesterol and HDL-cholesterol were measured, using enzymatic colorimetric assay (ADVIA Chemistry). Triglycerides were also measured by using enzymatic colorimetric assay (glycerol phosphate peroxidase) (ADVIA Chemistry), while LDL cholesterol was calculated by applying the Friedewald equation ( 24 ). The study questionnaire addressed sociodemographic factors, including age (continuous or categorical, such as 35 to 44, 45 to 54, 55 to 64, and 65 to 74 years of age) and sex, and the level of formal education (less than high-school, high-school and some college, and at least complete college or more. The study evaluated self-reported race/skin color as a social construct using the same question used in the Brazilian CENSUS (IBGE): “The Brazilian census (IBGE) describes people's color or race as “Black”, “Brown”, “White”, “Asian descendant” or “Brazilian indigenous”. If you were to answer the IBGE census today, how would you describe your own color or race?” The following response options were given: Black, Brown, White, Asian descendant, and Brazilian indigenous. All participants were asked about their use of prescription and nonprescription drugs and were requested to present their prescriptions, packages, package inserts and/or blister packs of the medications used in the last two weeks. All proceedings were performed in the same way at baseline and follow-up.

### Statistical analysis

The incidence of thyroid disease rates is expressed as percentage per year or as a cumulative incidence over a 4-year follow-up with respective 95% Confidence Intervals (95% CI). Categorical variables are reported as proportions and compared using the Chi-square test. Continuous variables with normal distribution are reported as means (Standard deviation, ±SD) and compared by analysis of variance (ANOVA), while those with non-normal distribution are presented as medians (Interquartile range, IQR) compared by means of the Kruskal-Wallis test. For showing differences among categories of each variable it was used Bonferroni post hoc test for categorical and continuous nonparametric variables.

Analysis were conducted using the Statistical Package for Social Sciences (SPSS) version 25. Confidence Intervals were calculated using the software R version 3.5.3 (R Core Team, Vienna, Austria).

## RESULTS

After exclusions ( Figure 1 ), 9,705 participants (median age, 50 (IQR, 45-57); 51.9% of women) with normal thyroid function at baseline examination that also have data about thyroid function over the 4-year follow-up were included in the incidence analysis. Over a 4-year follow-up, of the 9,705 participants, 9,079 remained with normal thyroid function (euthyroid), 17 developed incident overt hyperthyroidism, 49 incident subclinical hyperthyroidism, 377 incident subclinical hypothyroidism, and 183 incident overt hypothyroidism. Table 1 shows baseline sociodemographic and clinical characteristics of the 9,705 participants according to the incidence of thyroid disorders over a 4-year follow-up. Most cases of thyroid diseases were found in womencompared to men (P < 0.0001). Median age increases from overt hyperthyroidism to overt hypothyroidism. Frequency of thyroid diseases were different according to age-strata and race (respectively P < 0.0001 and P = 0.01).

**Table 1:** General characteristics of all 9,705 subjects according to the presence or not of thyroid function over a 4-year follow-up (2012-2014)

		Overt hyperthyroidism N = 17	Subclinical hyperthyroidism N = 49	Euthyroidism N = 9,079	Subclinical hypothyroidism N = 377	Overt hypothyroidism N = 183	All N = 9705	P
Age (years)**		49 (43.5-51)	52 (49-58)	50 (44-57)*^†^	53 (47-59)^†^	53 (46-60)*	50 (45-57)	<0.0001
Age-strata at baseline								<0.0001
	35-44	7 (41.2)*	8 (16.3)^†^	2766 (30.5)*^†‡^	80 (21.2)*^†‡^	41 (22.4)^‡^	2411 (24.8)	
	45-54	9 (52.9)	20 (40.8)	3611 (34.8)	148 (39.3)	73 (39.9)	3953 (40.7)	
	55-64	1 (5.9)	17 (34.7)	2093 (23.1)	112 (29.7)	45 (24.6)	2513 (25.9)	
	65-74	0(0)*	4 (8.2)^†^	609 (60.3)*^†‡^	37 (9.8)^‡^	24 (13.1)*^†‡^	828 (8.6)	
Women (%)		9 (52.9)*	35 (51.4)*	4665 (51.4)*	185 (49.1)*	142 (77.6)*	5036 (51.9)	<0.0001
Race (%)								0.01
	White	7 (41.2)*	30 (61.2)^†^	4552 (50.7)*^†‡^	230 (61.2)*^†‡^	109 (59.9)^‡^	4928 (51.4)	
	Mixed	5 (29.4)	11 (22.4)	2554 (28.5)	83 (22.1)	41 (22.5)	2694 (28.1)	
	Black	4 (23.5)	7 (14.3)	1525 (17)	54 (14.4)	21 (11.5)	1611 (16.8)	
	Asian	1 (5.9)	1 (2)	242 (2.7)	5 (1.3)	9 (4.9)	258 (2.7)	
	Indigenous	0	0	97 (1.1)	4 (1.1)	2 (1.1)	103 (1.1)	
Education (%)								0.74
	Less than high-school	3 (17.6)	4 (8.2)	1087 (12)	47 (12.5)	21 (11.5)	1162 (12)	
	High-school and some College	7 (41.2)	20 (40.8)	3249 (35.8)	132 (35)	55 (30.1)	3463 (35.7)	
	Complete College or more	7 (41.2)	25 (51)	4743 (52.2)	198 (52.5)	107 (58.5)	5080 (52.3)	
	Thyroid stimulating hormone (IU/ml)**	0.01 (0.01-1.63)*^#¥▪^	0.28 (0.10-0.34)^†‡^	1.79 (1.30-2.40)*^#♦α^	4.64 (4.24-5.22)^‡▪αβ^	2.53 (1.04-4.55)^†¥♦β^	1.84 (1.31-2.52)	<0.0001
Free-thyroxine (ng/ml)**		2.08 (1.28-2.90)^†‡^#	1.24 (1.12-1.36)*	1.18 (1.08-1.28)^‡^	1.15 (1.08-1.25)*^†^	1.24 (0.90-1.56)#	1.18 (1.08-1.29)	<0.0001
TPOAbs (%)**		17.1 (10.2-126.7)^‡^	14.61 (11.42-30.1)^†^	11.69 (11.15-14.86)*^†‡^	11.5 (8.75-16.5)^#^	13.19 (9.68-63.13)*^#^	11.19 (8.71-15.06)	<0.0001

**Median (Interquartile Range); For age: *Euthyroidism ≠ overt hypothyroidism; ^†^euthyroidism ≠ subclinical hypothyroidism; For age-strata 35-44 years of age, *overt hyperthyroidism, ^†^subclinical hyperthyroidism and ^‡^overt hypothyroidism ≠ from euthyroidism and subclinical hypothyroidism; for age-strata 65-74, *overt hyperthyroidism, ^†^subclinical hyperthyroidism and ^‡^subclinical hypothyroidism ≠ euthyroidism and overt hypothyroidism. For *women and for ^†^men, each category is ≠ all others; For self-reported skin color White *overt hyperthyroidism, ^†^subclinical hyperthyroidism and ^‡^overt hypothyroidism ≠ euthyroidism;and *^†‡^subclinical hypothyroidism. For TSH: *Overt hyperthyroidism ≠ euthyroidism (P <0.0001); ^†^Subclinical hyperthyroidism ≠ overt hypothyroidism (P<0.0001); ^‡^Subclinical hyperthyroidism ≠ subclinical hypothyroidism (P <0.0001); ^#^Overt hyperthyroidism ≠ euthyroidism (P = 0.002); ^¥^Overt hyperthyroidism ≠ overt hypothyroidism; ^▪^Overt hyperthyroidism ≠ subclinical ypothyroidism (P <0.0001); ^♦^Overt hypothyroidism ≠ euthyroidism (P <0.0001); ^α^Subclinical hypothyroidism ≠ euthyroidism (P <0.0001); ^β^Overt hypothyroidism ≠ subclinical hypothyroidism; For FT4: *Subclinical hyperthyroidism ≠ subclinical hypothyroidism (P = 0.045); ^†^Overt hyperthyroidism ≠ subclinical hypothyroidism (<0.0001); ^‡^Overt hyperthyroidism ≠ euthyroidism (P <0.0001); ^#^Overt hyperthyroidism ≠ overt hypothyroidism, (P = 0.002); For TPOAb: *Overt hypothyroidism ≠ euthyroidism (P <0.0001); ^†^Subclinical hyperthyroidism ≠ euthyroidism (P <0.0001); ^‡^Overt hyperthyroidism ≠ euthyroidism (P = 0.046); ^#^Overt hypothyroidism ≠ subclinical hypothyroidism (P = 0.01).

Table 2 shows an annual incidence of thyroid diseases according to sex, age-strata, and self-reported race. The incidence of overt hyperthyroidism was similar for men and women. The incidence of subclinical hyperthyroidism and overt hypothyroidism was higher in women when compared to men. However, the incidence of subclinical hypothyroidism is discretely higher in men when compared to women (1.11% in men vs. 0.99% in women but the difference was not statistically significant, P = 0.38). Considering all dysfunctions together, the women:men ratio for thyroid diseases was 1.36.

**Table 2:** Annual incidence (95% Confidence Interval – 95% CI) of thyroid diseases expressed in percentages according to sex, age-strata at baseline and self-reported race

	Overt hyperthyroidism N = 17	Subclinical hyperthyroidism N = 49	Subclinical hypothyroidism N = 377	Overt hypothyroidism N = 183	All thyroid diseases	P
Sex (%)						<0.0001
Women	0.052*	0.19*	0.99*	0.76*	1.99	
	0.01-0.19	0.09-0.38	0.74-1.32	0.54-1.06	0.12-14.6	
Men	0.047^†^	0.082^†^	1.11^†^	0.24^†^	1,48	
	0.01-0.18	0.02-0.24	0.83-1.47	0.13-0.45	(0-11.93)	
All	0.048	0.14	1.03	0.51	1.73	
	0.02- 0.13	0.08-0.25	0.84-1.26	0.38-0.68	1.48- 2.01	
Age-strata (years)						
35-44	0.056*	0.09^†^	0.73*^†‡^	0.41^‡^		<0.0001
	0.01-0.30	0.02-0.35	0.44-1.19	0.21-0.79		<0.0001
45-54	0.07	0.12	0.98	0.47		
	0.02-0.24	0.04-0.31	0.70-1.36	0.29-0.76		
55-64	0.02	0.21	1.25	0.55		
	0-0.24	0.08-0.52	0.86-1.80	0.31-0.96		
65-74	0*	0.17^†^	1.50^‡^	0.90*^†‡^		
	0-0.64	0.02-0.92	0.81-1.09	0.39-1.93		
Self-reported race (%)						P = 0.01
White	0.04*	0.17^†^	1.26*^†‡^	0.61^‡^		
	0.01-0.17	0.08-0.35	0.97-1.63	0.42-0.89		
Mixed	0.050	0.11	0.80	0.40		
	0-0.27	0.03-0.36	0.51-1.25	0.21-0.76		
Black	0.067	0.12	0.87	0.35		
	0-0.43	0.02-0.51	0.49-1.50	0.13-0.84		
Asian	0.10	0.10	0.91	0.52		
	0-2.11	0-2.11	0.18-3.34	0.05-2.76		
Indigenous	0	0	1.01	0.52		
	0-4.75	0-4.75	0.06-6.21	0-5.52		

Annual incidence expressed in percentages. For *women and ^†^men: each groups is ≠ from the others. For age-strata 35-45, *overt hyperthyroidism, ^†^subclinical hyperthyroidism and ^‡^overt hypothyroidism are ≠ from euthyroidism and *^†‡^subclinical hypothyroidism; for age-strata 65-74, *overt hyperthyroidism, ^†^subclinical hyperthyroidism and ^‡^subclinical hypothyroidism ≠ euthyroidism and *^†‡^overt hypothyroidism; For self-reported skin color: white *overt hyperthyroidism, ^†^subclinical hyperthyroidism and ^‡^overt hypothyroidism ≠ euthyroidism and subclinical *^†‡^subclinical hypothyroidism.

**Figure 2:**
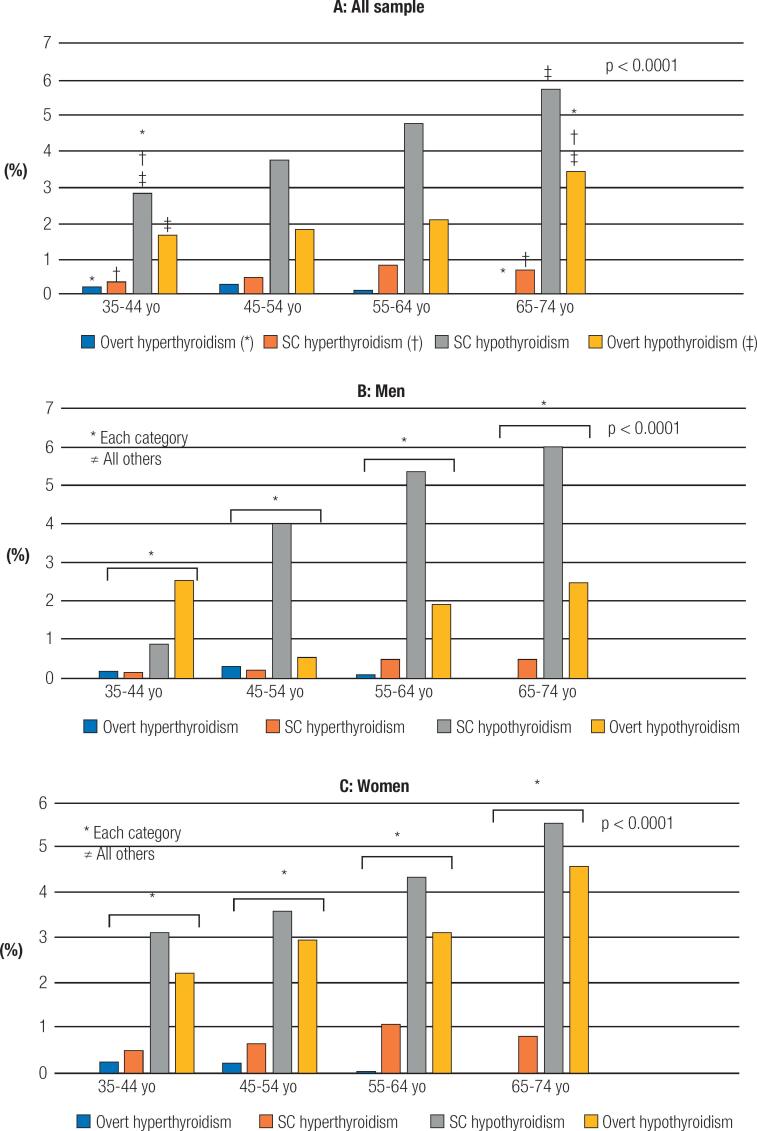
Cumulative incidence of thyroid diseases according to age-strata (yo = years of age).

Figure 2 shows the cumulative incidence of thyroid diseases according to age-strata in the entire sample by sex. The results showed a cumulative incidence of thyroid disease of 6.7% (1.73% per year): 0.19% for overt hyperthyroidism (0.048% per year), 0.54% for subclinical hyperthyroidism (0.14% per year), 1.98% for overt hypothyroidism (0.51% per year), and 3.99% for subclinical hypothyroidism (1.03% per year). In the entire sample, the incidence of hypothyroidism is higher than for hyperthyroidism. The incidence of hypothyroidism is also higher in the older-age strata for entire sample, including both men and women while incidence of hyperthyroidism was higher in the younger age-strata. As expected, the incidence of thyroid diseases is higher in women when compared to men. However, the women:men ratio for overt hyperthyroidism, subclinical hyperthyroidism, and overt hypothyroidism and subclinical hypothyroidism were, respectively, 1.1, 2.3, 0.9 e 3.2.

Of the 17 incident cases of hyperthyroidism, 9 (52.9%) were identified based on TSH and FT4 levels and 8 (47.1%) were identified by use of medication. Of them, 9 (52.9%) were women and 7 of them (77.8%) were under treatment. Of the 8 men with overt hyperthyroidism, only 1 (12.5%) were under treatment. Mean, median and range of participants treated with thiamazole were respectively 7.5 (±1.0), 7.5 ( 5 - 10 ), and range of 5 to 10 mg. Of the 183 incident cases of hypothyroidism, 49 (26.8%) were identified based on TSH and FT4 levels and 134 (73.2%) were identified by use of medication. Of them, 142 (77.6%) were women and 113 (79.6) of them where under treatment. However, of the 41 men with overt hypothyroidism, only 21 (51.2%) were under treatment with levothyroxine. (P < 0.0001). Mean, median and range of levothyroxine in participants treated with levothyroxine were respectively 90 mcg (±14.7), 75 mcg (38-100) with a range from 25 to 143 mcg.

**Figure 3:**
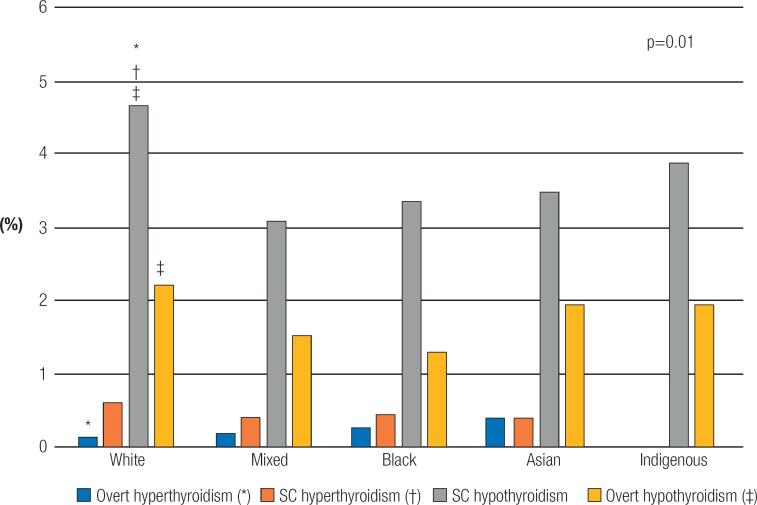
Cumulative incidence of hyperthyrodism and hypothyroidism according to self-reported race.

The incidence of overt hyperthyroidism is the highest in participants who self-reported themselves Asians (0.10%, 95% CI, 0.2-1.1) followed by Blacks (0.067%, 95% CI, 0-0.43) Mixed (0.05%, 95% CI, 0-0.27) and Whites (0.04%, 95% CI 0.01-0.17). The incidence of overt hypothyroidism was higher in Whites 0.61%, 95% CI, 0.42-0.89), followed by Asians (0.52, 95% CI, 0.05-2.76), and Indigenous (0.52%, 95% CI,0.05-2.76) with the lower and similar incidences for Mixed (0.40%, 95% CI, 0.21-0.76) and Blacks (0.35%, 95% CI, 0.13-0.84). Participants who self-reported themselves as Mixed race presented an incidence of overt hyperthyroidism and overt hypothyroidism that is intermediary between Whites and Blacks ( Table 2 ). Figure 3 shows the cumulative incidence of thyroid dysfunction according to self-reported race.

**Figure 4:**
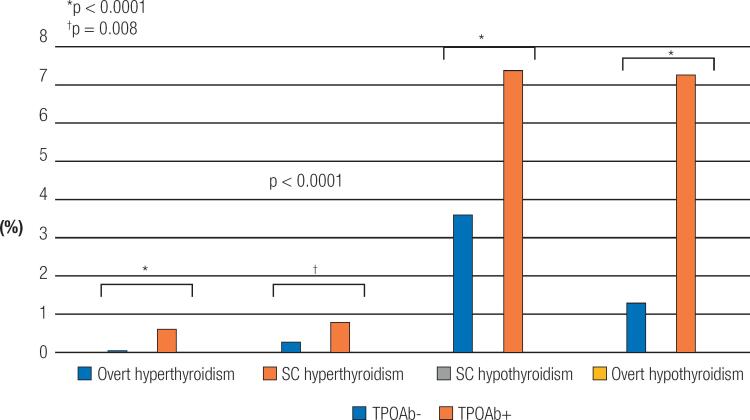
Incidence of overt and subclinical hyperthyroidism and hypothyroidism according to the presence or not of TPO antibodies (TPOAb) at baseline.

Figure 4 shows the incidence of thyroid diseases over a 4-year follow-up according to the presence or not of TPOAb at baseline. Incidence of thyroid diseases is always higher in participants with positive TPOAb, when compared to participants with no TPOAb, at baseline. The ratio of thyroid diseases in participants with positive TPOAb, when compared to participants with negative TPOAb, at baseline for overt hyperthyroidism, subclinical hyperthyroidism, subclinical hypothyroidism, and overt hypothyroidism were 5.6, 2.7, 2.3, and 5.2, respectively.

## DISCUSSION

The present study showed, for the first time, a high annual and cumulative incidence of new overt and subclinical hyperthyroidism and hypothyroidism in a large Brazilian cohort after a 4-year follow-up. The results highlighted a higher incidence of hypothyroidism in the country, but a similar incidence of hyperthyroidism compared to other countries worldwide. Our data also presented a lower women:men ratio for thyroid diseases compared to other classical studies that evaluate thyroid function. Incidence of overt hypothyroidism, but not of overt hyperthyroidism increased with ageing. Incidence of thyroid diseases was higher in participants with positive TPOAb at baseline compared to other participants.

The overall incidence of thyroid dysfunctions was higher in women, when compared to men, as demonstrated by the women:men ratio of 1.36, but lower than the ratios reported by classical studies on the incidence or prevalence of thyroid diseases ( 13 , 25 ). Frequency of treatment of overt thyroid diseases is higher in women compared to men. The presence of TPOAb antibodies was associated with a higher incidence of all thyroid diseases. Regarding treatment, more women than men are under treatment for thyroid diseases even considering a sample with more access to health care compared to the general population in Brazil. This low frequency of treatment in men confirmed a previous study in a population-based sample of older men and women ( 16 ).

A meta-analysis of seven studies in Europe reported an incidence of 226.2 (222.26-230.17 per 100,000) for hypothyroidism ( 3 ). These numbers were lower than Brazilian data if our results were expressed per 100,000 per year (for overt hypothyroidism in ELSA-Brasil of 511.87/100,000). Another important point is that, although we showed a higher incidence in women when compared to men, the women:men ratio for overt hypothyroidism is 3.2 which is lower than the women:men ratio in this European meta-analysis of 5.1 for overt hypothyroidism ( 3 ). The low women:men ratio was previously reported in the prevalence of thyroid diseases at the ELSA-Brasil baseline ( 18 ) as well as in a population-based sample of older adults with a diagnosis of thyroid diseases in the city of São Paulo ( 16 ).

For overt hyperthyroidism, the European meta-analysis reported an incidence of 51 (49.23-52.88) for hyperthyroidism, which is similar to the incidence in ELSA-Brasil if it were expressed by 100,000 (48.42/100,000). Once again, the women:men ratio for overt hyperthyroidism of 1.1 was lower than the women:men ratio in the meta-analysis of 5.1.

Gopinath and cols. evaluated the 5-year incidence of thyroid dysfunction in a sample of older adults (≥ 55 years) in Australia, reporting an incidence of all thyroid diseases of 4.7% compared to 6.7% in Brazil. ( 26 ) As the incidence of hyperthyroidism was similar in both countries, the difference in the percentage of thyroid diseases is related to a higher incidence of hypothyroidism in Brazil compared to Australia, even considering that the median age of our sample is younger than that in Gopinath's study (mean age in Gopinath's study: 67.6 ± 7.6; our study: 51.2 ± 8.9) and our follow-up is shorter (4 years compared to 5 years in the Australian study. The results from the present study regarding high incidence of hypothyroidism and a similar incidence of hyperthyroidism when compared to some other countries are in accordance with previous studies that showed a lower incidence of hyperthyroidism and a higher incidence of hypothyroidism in places with higher levels of iodine intake (6,27,28), such as the case of Brazil. The presence of goiter was very high in the past and decreased after the iodination of salt, which began in 1953. Since the beginning of the twenty-first century, the discussion about salt iodination in Brazil has become a matter of intense debate. A meta-analysis of seven studies from the Southeast region of Brazil showed great heterogeneity among studies, with no available data from other Brazilian regions ( 29 ). More recently, results from the PNAISAL ( 30 ) (National Research to Evaluate the Impact of Salt Iodination), a national research in Brazil including 18,978 schoolchildren from all regions of the country, conducted to evaluate the iodine nutritional status in the Brazilian population classified Brazil as a country with a more-than-adequate iodine intake.

Incidence of hypothyroidism increased with age as expected. However, not in the same pace as studies in countries with a high prevalence of olders. (25,26,28,31) Our data is in agreement with data from another low- and middle-income country that also showed a moderate increase of hypothyroidism with age ( 4 ). For overt hyperthyroidism, our data showed a higher incidence in the younger age-strata. As the number of participants in the younger age-strata is greater than in the older age-strata and the incidence of overt hyperthyroidism is lower than for hypothyroidism, in a sample of middle-aged adults, it is more difficult to show the increase with ageing. Our data is similar to data from another low- and middle-income country that also did not show an increase in the incidence of overt hyperthyroidism with ageing ( 4 ). In addition, Brazil has still a higher early mortality especially for cardiovascular diseases compared to high-income which difficult to show increasing trends in old-age for several variables.

The ELSA-Brasil study examined a highly admixed population, with a considerable proportion of participants that classified themselves as Mixed race. Although race/skin color in ELSA-Brasil was measured using the question of the Brazilian census as a social construct, results of higher incidence of hypothyroidism in whites and hyperthyroidism in blacks are in accord with previous studies that measure race/skin-color in different ways ( 32 - 34 ). However, it is important to notice that the highest incidence of overt hyperthyroidism was detected in Japanese descendants and for hypothyroidism Asians was in the second place after Whites. This was in agreement with a previous study of Sgarbi et al. with Brazilian Japanese ( 14 ) but not with the prevalence/incidence of thyroid diseases in Japan ( 35 , 36 ) that is much lower, suggesting the influence of environment and lifestyle more than genetic inheritance to explain results in Japanese descendants in Brazil. Results for the incidence of overt hypothyroidism in the Indigenous category have to be interpreted with caution because of the small number in the sample.

Our results showed that euthyroid individuals with positive TPOAb presented a higher incidence of overt and subclinical hyperthyroidism and hypothyroidism than TPOAb negative participants. Our results are in accord with previous published studies. ( 37 - 40 ) For incident overt hypothyroidism and hyperthyroidism the risk of participants TPOAb positive is 5.2 and 5.6 times higher compared to participants with negative TPOAb respectively. The ratio of conversion for overt thyroid diseases were higher than for subclinical thyroid diseases. A recent analysis about the prevalence of TPOAb in Brazil using data from ELSA-Brasil study showed values in accordance with a country with adequate iodine intake ( 41 ). It would be interesting to monitor TPOAb levels and incidence of thyroid diseases using data from the third data collection over a longer follow-up.

Our study has some limitations. ELSA-Brasil is not a population-based study. However, it is a multicentric cohort study in six different cities located in three different regions of the country: South, Southeast, and Northeast. The study includes a sample with higher education and higher average monthly net family income compared with the general population of Brazil. Although these differences between ELSA-Brasil participants and general population in Brazil, the social and ethnic diversity of the cohort is similar to the heterogeneous populations of mostly low- and middle-income people living in large cities in Brazil. This suggests that our external validity may extend to urban centers with similar characteristics both inside and outside of Brazil. Furthermore, there are several similarities in the prevalence of selected behavioral risk factors and chronic conditions, as these have been assessed with similar procedures in the ELSA-Brasil and in the Surveillance System of Risk and Protection Factors for Chronic Diseases by Telephone Survey (VIGITEL), an annually performed telephone-based behavioral risk factor survey, producing representative data for adults living in Brazil's 27 state capitals and the Federal District ( 42 ). Overt hypothyroidism was defined based on TSH and FT4 levels and the use of levothyroxine. However, levels of TSH and FT4 were measured only one time, not considering the great intra-individual variability of TSH values and some occasionally variations of TSH levels may be interpreted erroneously as permanent disease. At baseline of the ELSA-Brasil, a high use of levothyroxine was reported in the sample, especially in women with a higher average monthly family net income, when compared to others. Therefore, both problems may contribute to some degree of misclassification, especially regarding subclinical and overt hypothyroidism in women in the present analysis.

This study also has some strengths. Thyroid function at baseline and after a 4-year follow-up were measured, which allowed us to determine the incidence of thyroid diseases in Brazil for the first time in a large and multicentric sample. Although the higher frequency of women, the study included a similar number of men. Study protocols were exactly the same for all centers, and data collection was performed under strict quality control. These results in a highly admixed population bring original information about the incidence of thyroid diseases in participants that self-reported themselves as Mixed, a group that, except for a previous study in Brazil ( 17 ), had never been analyzed in other studies.

In conclusion, results from ELSA-Brasil showed that the high incidence of hypothyroidism may well be compatible with a country with a more-than-adequate iodine intake. Incident data also showed a low women:men ratio for thyroid diseases and a lower proportion of treatment in men with overt thyroid diseases compared to women. Both data highlight the importance of the diagnosis and treatment of thyroid diseases among men in Brazil.
